# miR‐21‐5p promotes cell proliferation and G1/S transition in melanoma by targeting CDKN2C

**DOI:** 10.1002/2211-5463.12819

**Published:** 2020-03-24

**Authors:** Zhaohui Yang, Bo Liao, Xiaoyan Xiang, Sha Ke

**Affiliations:** ^1^ Department of Burns and Plastic Surgery Affiliated Hospital of North Sichuan Medical College Nanchong China; ^2^ Department of Urology Affiliated Hospital of North Sichuan Medical College Nanchong China; ^3^ Department of Neurology Affiliated Hospital of North Sichuan Medical College Nanchong China

**Keywords:** melanoma, CDKN2C, G0/G1 phase arrest, proliferation, miR‐21‐5p

## Abstract

Human melanoma is a highly malignant tumor originating from cutaneous melanocytes. The noncoding RNA microRNA (miR)‐21‐5p has been reported to be expressed at high levels in malignant melanocytic skin tissues, but its potential functional role in melanoma remains poorly understood. Here, we explored the cellular effects of miR‐21‐5p on melanoma *in vitro* and the underlying mechanisms. Quantitative real‐time PCR was used to show that miR‐21‐5p is significantly up‐regulated in clinical samples from patients with melanoma as compared with adjacent noncancerous tissues. Overexpression of miR‐21‐5p significantly enhanced, whereas knockdown attenuated, cell proliferation and G1/S transition in melanoma cell lines (A375 and M14). Luciferase reporter assays were used to show that the cyclin‐dependent kinase inhibitor 2C (*CDKN2C*) is a downstream target of miR‐21‐5p. Furthermore, miR‐21‐5p mimics resulted in a decrease in CDKN2C expression, and *CDKN2C* expression was observed to be inversely correlated with miR‐21‐5p expression in melanoma tissues. Rescue experiments were performed to show that overexpression of *CDKN2C* partially reversed the effects of miR‐21‐5p up‐regulation on A375 cells. Consistently, knockdown of CDKN2C abolished the effects of miR‐21‐5p down‐regulation on A375 cells. Overall, our studies demonstrate that miR‐21‐5p can promote the growth of melanoma cells by targeting *CDKN2C*, which may induce G0/G1 phase arrest of melanoma cells.

## Graphical abstract

miR‐21‐5p down‐regulates *CDKN2C* expression, thereby promoting G1/S transition and enhancing cell proliferation in melanoma.

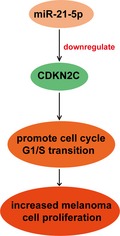

**Abbreviations**
CCK‐8: Cell Counting Kit‐8
CDKI: cyclin‐dependent kinase inhibitor
CDKN2C: cyclin‐dependent kinase inhibitor 2C
miR: microRNA
MUT: mutant
NC: negative control
SD: standard deviation
WT: wild‐type

Melanoma is thought as the most aggressive form of human cutaneous neoplasm with markedly increased incidence in recent decades [1]. Currently, surgical intervention for early‐stage disease and systemic chemotherapy for locally advanced disease have been the mainstays of treatments for patients with melanoma [2, 3]. Although there is a certain beneficial effect, the clinical outcome remains unsatisfactory for patients, with a 10‐year survival rate still less than 10% [4]. With the development of molecular biology, it provides a great possibility for us to investigate the precise molecular mechanism underlying melanoma pathogenesis.

MicroRNAs (miRs) are small (22–26 nucleotides), noncoding RNAs that can regulate gene expression primarily by recognizing and binding to the potential targeting sites in the 3′ UTR of target mRNAs, and thereby are involved in diverse biological processes [5, 6]. One possible mechanism underlying melanoma pathogenesis is altered expression of the miRNA expression profile. For example, miR‐373 expression was reported to be highly up‐regulated in melanoma tissues and able to promote cell migration by negatively targeting salt‐inducible kinase 1 [7]. Noori *et al.* [8] demonstrated that miR‐30a inhibits melanoma tumor metastasis by targeting ZEB2 and E‐cadherin. Recent studies have highlighted the importance of miR‐21‐5p in tumor progression specifically in the process of cell proliferation, including non‐small lung cancer [9], colon cancer [10] and ovarian cancer [11]. Interestingly, increased miR‐21 expression has been observed during the transition from a benign melanocytic lesion to malignant melanoma [12]. Similarly, miR‐21‐5p has been reported to be overexpressed in malignant melanocytic skin tissues compared with benign tumors by global miRNA profiling [13], as well as next‐generation sequencing by Babapoor *et al.* [14] and Latchana *et al.* [2]. Functionally, miR‐21 has been demonstrated to enhance the invasiveness of melanoma cells by inhibition of tissue inhibitor of metalloproteinases 3 [15]. However, how miR‐21‐5p regulates cell proliferation in melanoma cells remains poorly understood.

Accumulating evidence indicates that deregulation of cell cycle at G1/S restriction point (cell growth), S phase (DNA replication) and G2/M phase (mitosis) are considered as critical issues among virtually all types of human tumors, including melanoma [16]. Cyclin‐dependent kinase inhibitor 2C (*CDKN2C*, also named p18) is an important cell‐cycle regulator and cyclin‐dependent kinase inhibitor (CDKI) [17, 18]. Despite a negative regulatory role of *CDKN2C* in G0/G1 cell‐cycle progression, the functional role of *CDKN2C* in cell‐cycle regulation still remains unknown in melanoma.

The main objective of this study was to reveal the regulatory role of miR‐21‐5p and *CDKN2C* in melanoma cell proliferation and cell‐cycle progression. Furthermore, we further evaluated whether miR‐21‐5p regulates cell proliferation and cell cycle by directly targeting *CDKN2C* in melanoma cells. Our study may provide new evidence to elucidate the role of miR‐21‐5p and *CDKN2C* in melanoma progression.

## Materials and methods

### Tissue samples

Melanoma tissues, along with matched adjacent tissues, were collected from 20 patients with melanoma who underwent surgical resection from Affiliated Hospital of North Sichuan Medical College. This cohort included 12 male and 8 female patients, with a median age of 52 years (range: 34–72 years). These 20 patients with melanoma were diagnosed as stage I (*n* = 5), II (*n* = 8) or III (*n* = 7) according to the consensus of the tumor node metastasis staging system. Before surgery, the patients were confirmed to not receive any antitumor treatment. Collected fresh tissue samples were snap frozen in liquid nitrogen with subsequent storage at −80 °C until use. We obtained written informed consent from all patients. The study protocol conformed to the ethical guidelines outlined in the Declaration of Helsinki and was approved by the research ethics committee of Affiliated Hospital of North Sichuan Medical College (approval no. 2014121M).

### Cell culture

Two human melanoma cell lines, A375 and M14, were purchased from ATCC (Manassas, VA, USA) and cultured according to the supplier’s information in Dulbecco’s modified Eagle’s medium (Gibco, Rockville, MD, USA) supplemented with 10% FBS (Gibco), which were both maintained in a humidified atmosphere containing 5% CO_2_ at 37 °C.

### Oligonucleotide transfection

The miR‐21‐5p mimic, inhibitor and their negative controls (NCs) were purchased from GenePharma Co. Ltd. (Shanghai, China). Small interference RNA for *CDKN2C* (si‐CDKN2C) and its NC (si‐NC) were also synthesized by GenePharma Co. Ltd. The *CDKN2C* overexpression plasmid was constructed by inserting CDKN2C cDNA into a pcDNA3.1 vector by GenePharma Co. Ltd. For transfection, A375 or M14 cells were seeded at a density of 2 × 10^5^ cells per well in a six‐well culture dish, and transfection was performed when 80% confluence was achieved. A total of two 8‐µL (500 ng·µL^−1^) plasmids (pcDNA3.1‐CDKN2C, si‐CDKN2C) or mimics (miR‐21‐5p mimic, inhibitor and NC) and 8 µL Lipofectamine 2000 (Invitrogen, Carlsbad, CA, USA) were suspended in 100 µL Opti‐MEM (Gibco), and the final concentration of *CDKN2C* plasmids or mimics used was 1 mg·mL^−1^. Then, the mixture was added into the cell culture and incubated for 48 h.

### Quantitative real‐time PCR

After using TRIzol reagent (Invitrogen) extraction to obtain RNA from tissues and cell lines, we reverse transcribed the RNA samples into cDNA by M‐MLV RT kit (Promega Corporation, Madison, WI, USA). The quantitative real‐time PCR was done using iQ SYBR Green Supermix Kit (Bio‐Rad, Hercules, CA, USA) on a 7900HT Fast Real‐Time PCR system (Thermo Fisher Scientific, Inc., Waltham, MA, USA). The PCR procedure condition was as follows: preheating step of 10 s at 95 °C, followed by denaturation at 95 °C for 5 s for 40 cycles, and annealing and extension at 60 °C for 20 s. According to Livak and Schmittgen’s method [19], relative gene expression was calculated using the $2^{-\Delta \Delta C_{t}}$ values.

### Cell proliferation assay

The proliferation of A375 and M14 cells was evaluated by Cell Counting Kit‐8 (CCK‐8) detection kit (Beyotime Biotechnology, Shanghai, China). In brief, the transfected cells were seeded at 3 × 10^3^ cells per 6.4‐mm dish and cultured overnight. At every 24 h, 10 μL CCK‐8 solutions was put in each well, and cells were incubated at 37 °C for another 2 h. To determine the absorbance (*A*) values of each well, we measured absorbance at 450 nm using a microplate reader (Thermo Fisher Scientific).

### Cell‐cycle analysis

After 48‐h transfection, cell‐cycle distribution was analyzed by Cell Cycle Detection Kit (KeyGen Biotech, Nanjing, China). In brief, cells were collected and fixed in 70% ethanol overnight at 4 °C. Following washing with PBS, cells were treated with RNase A (10 U·mL^−1^) and stained with propidium iodide (50 μg/mL) at room temperature for 30 min. Then, the mixture samples were measured using BD FACSCalibur flow cytometer equipped with modfit Software (BD Biosciences, San Jose, CA, USA). The proportion of cells in the G0/G1, S and G2/M phases was quantified in each group.

### Plasmid construction and luciferase activity assay

The potential binding sequence of miR‐21‐5p and *CDKN2C* was predicted by TargetScan (http://www.targetscan.org/vert_71/). For luciferase activity assay, the wild‐type (WT) and mutant type (MUT) 3′ UTR sequences of *CDKN2C* were amplified and constructed into psiCHECK2 vector (Promega). Then, 1 μg WT or MUT plasmids and mimic or NC were cotransfected into HEK293T cells (5 × 10^4^) using Invitrogen Lipofectamine 2000. Forty‐eight hours posttransfection, luciferase activity was evaluated using a dual‐luciferase reporter assay kit (Promega).

### Western blot

Total cellular protein was isolated using radioimmunoprecipitation assay lysis buffer (Beyotime Biotechnology). Thirty micrograms of protein was separated by 10% SDS/PAGE and then transferred onto poly(vinylidene difluoride) membranes (Millipore, Billerica, MA, USA). After blocking membranes with fat‐free milk in TBS with 0.1% Tween 20 for 2 h at room temperature, the proteins were probed with specific primary antibodies against p18 and glyceraldehyde‐3 phosphate dehydrogenase, and then incubated with horseradish peroxidase‐conjugated secondary antibody (Santa Cruz Biotechnology, Dallas, TX, USA) at room temperature for 1 h. The protein bands were visualized with an enhanced chemiluminescence solution (Thermo Fisher Scientific).

### Statistical analysis

All of the *in vitro* experiments were performed at least in triplicate. Each value was expressed as mean ± standard deviation (SD). The statistical software package spss version 19.0 software (SPSS Inc., Chicago, IL, USA) was used for data analysis. The correlation between miR‐21‐5p and *CDKN2C* was measured by Spearman’s correlation analysis. Comparison between two groups and multiple groups, the Student’s *t*‐test and one‐way ANOVA followed by Dunnett’s post hoc test or Tukey’s test were carried out. A *P* value of less than 0.05 is considered to be statistically significant.

## Results

### miR‐21‐5p was negatively associated with *CDKN2C* mRNA expression in melanoma tissues

**Fig. 1:**
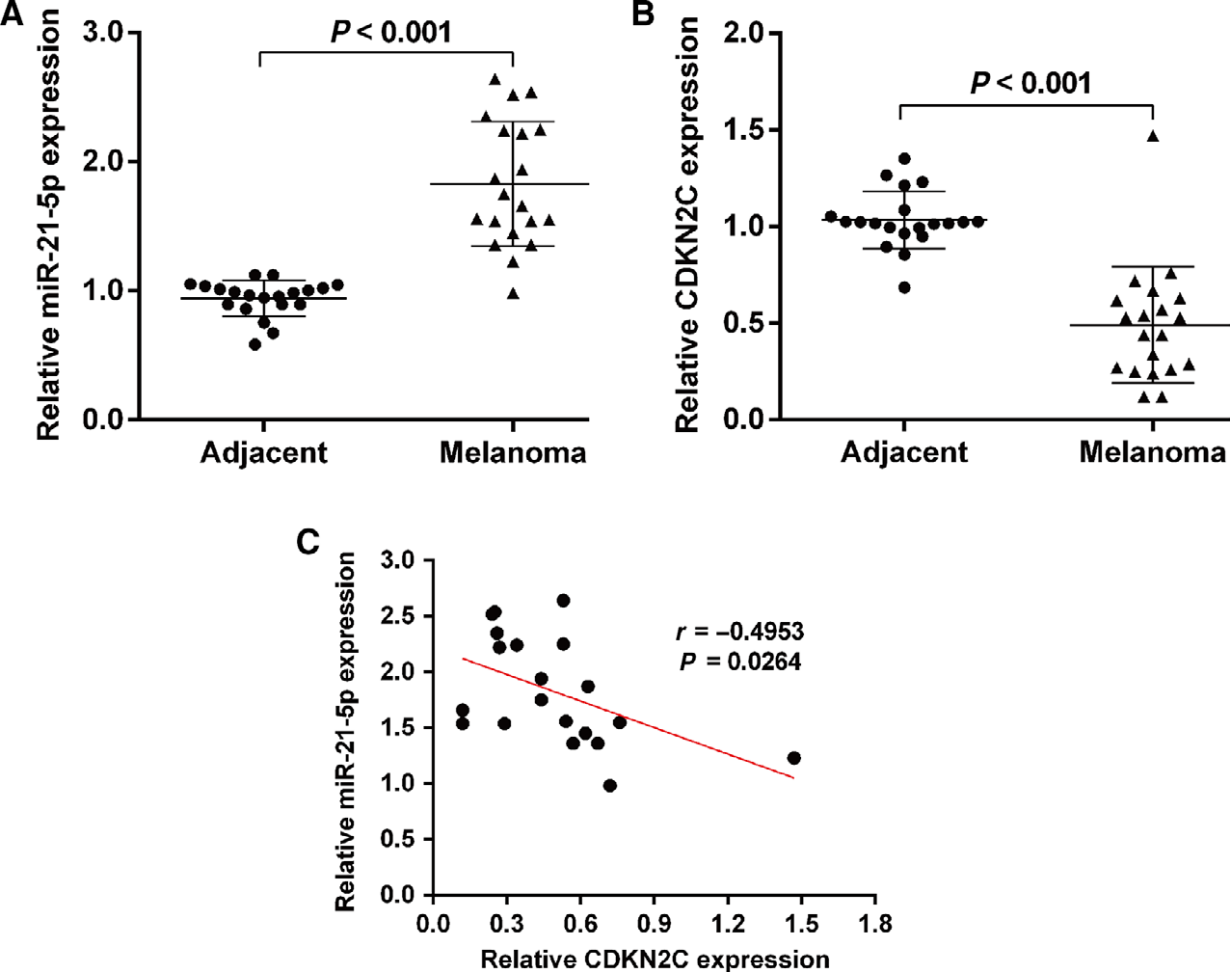
miR‐21‐5p was negatively associated with *CDKN2C* mRNA expression in melanoma tissues. (A) Quantitative real‐time PCR analysis of miR‐21‐5p levels in 20 melanoma tissues and matched adjacent tissues showing that miR‐21‐5p expression was increased in melanoma tissues. (B) Quantitative real‐time PCR analysis of *CDKN2C* mRNA levels in 20 melanoma tissues and matched adjacent tissues showing that *CDKN2C* expression was decreased in melanoma tissues. (C) Spearman’s correlation analysis for the association between miR‐21‐5p levels and *CDKN2C* mRNA levels in melanoma tissues.

To investigate the potential biological role of miR‐21‐5p in cell proliferation of melanoma, we analyzed the expression profile of miR‐21‐5p in 20 paired melanoma tissues and matched adjacent tissues using quantitative real‐time PCR. From Fig. 1A, we can see that the expression of miR‐21‐5p was significantly increased in melanoma tissues compared with adjacent tissues. Meanwhile, we determined the expression of *CDKN2C* mRNA in these tissues. As shown in Fig. 1B, *CDKN2C* mRNA levels were notably down‐regulated in melanoma tissues in comparison with adjacent tissues. Furthermore, Spearman’s correlation analysis further demonstrated that the expression of miR‐21‐5p was inversely correlated with the *CDKN2C* mRNA levels in melanoma tissues (*P* = 0.0264; Fig. 1C).

### miR‐21‐5p promoted cell proliferation and cell‐cycle G1/S transition in melanoma cells

**Fig. 2:**
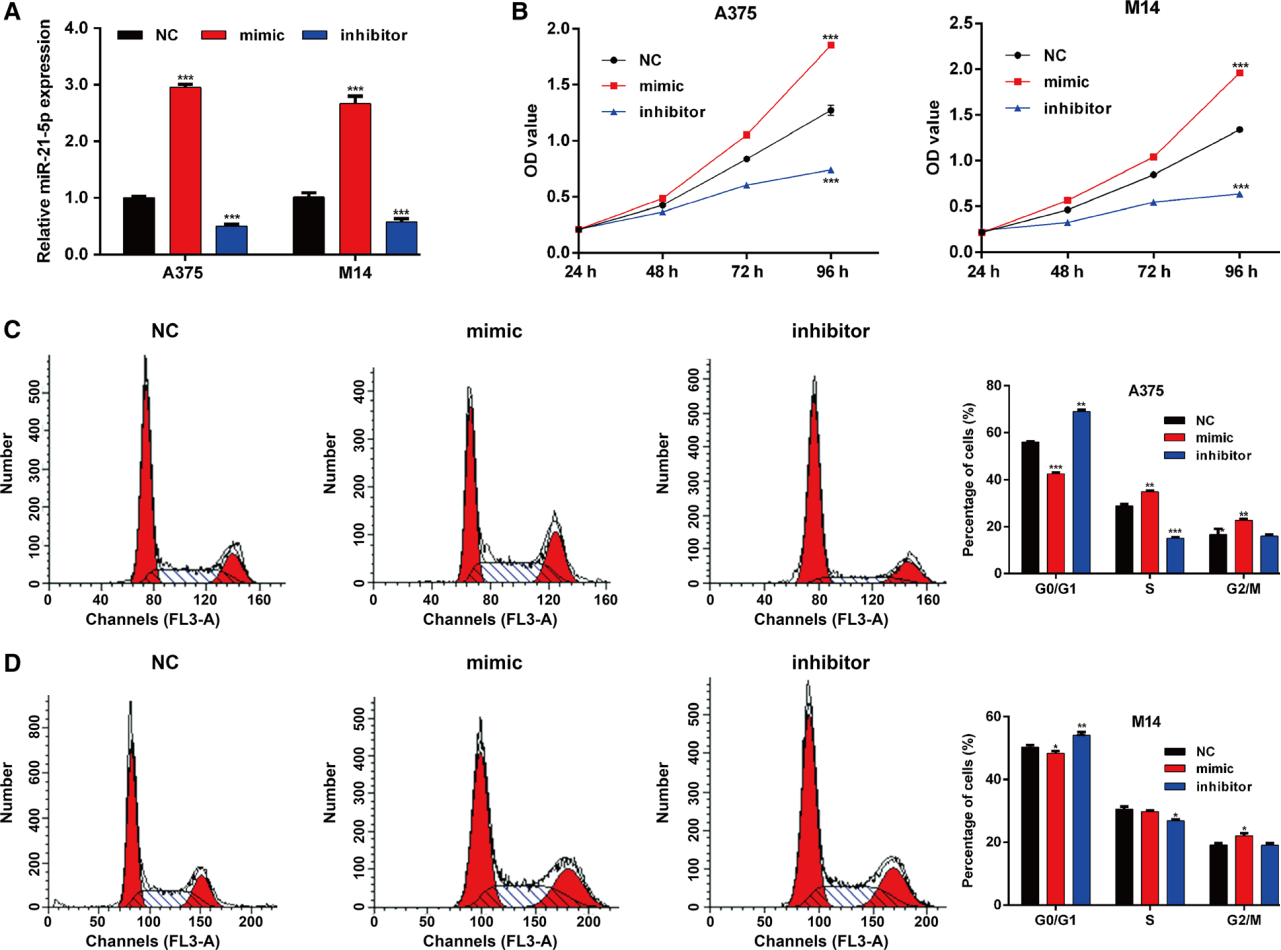
miR‐21‐5p promoted cell proliferation and cell‐cycle progression in melanoma cells. (A) The relative expression of miR‐21‐5p was measured by quantitative real‐time PCR analysis of A375 and M14 cells transfected with miR‐21‐5p mimic, inhibitor or NC for 48 h. (B) The cell proliferation rate was measured by CCK‐8 assay every 24 h. (C, D) Flow cytometry was performed to detect cell‐cycle distribution in A375 and M14 cells. Each sample was analyzed at least three times. Data were expressed as mean ± SD. Differences were evaluated using one‐way ANOVA, followed by Dunnett’s test. *P < 0.05, **P < 0.01, ***P < 0.001, compared with NC.

The significantly increased miR‐21‐5p expression in melanoma tissues leads us to make a hypothesis that miR‐21‐5p might be an oncogene in melanoma. To confirm this hypothesis, we transfected two melanoma cell lines (A375 and M14) with miR‐21‐5p mimic and inhibitor. As shown in Fig. 2A, miR‐21‐5p expression was significantly up‐regulated in the miR‐21‐5p mimic‐transfected A375 and M14 cells, but down‐regulated in miR‐21‐5p inhibitor‐transfected A375 and M14 cells. Next, CCK‐8 assay showed that overexpression of miR‐21‐5p remarkably enhanced cell proliferation, whereas knockdown of miR‐21‐5p inhibited cell proliferation in both A375 and M14 cells (Fig. 2B). Moreover, flow cytometry results showed the proportion of G0/G1 cells in A375 (Fig. 2C) and M14 (Fig. 2D) cells was significantly decreased after miR‐21‐5p mimic transfection, whereas the proportion of G0/G1 cells was strikingly increased after miR‐21‐5p inhibitor transfection compared with NC transfection. These data suggest that miR‐21‐5p could promote proliferation and cell‐cycle G1/S transition.

### *CDKN2C* was a target gene of miR‐21‐5p in melanoma

**Fig. 3:**
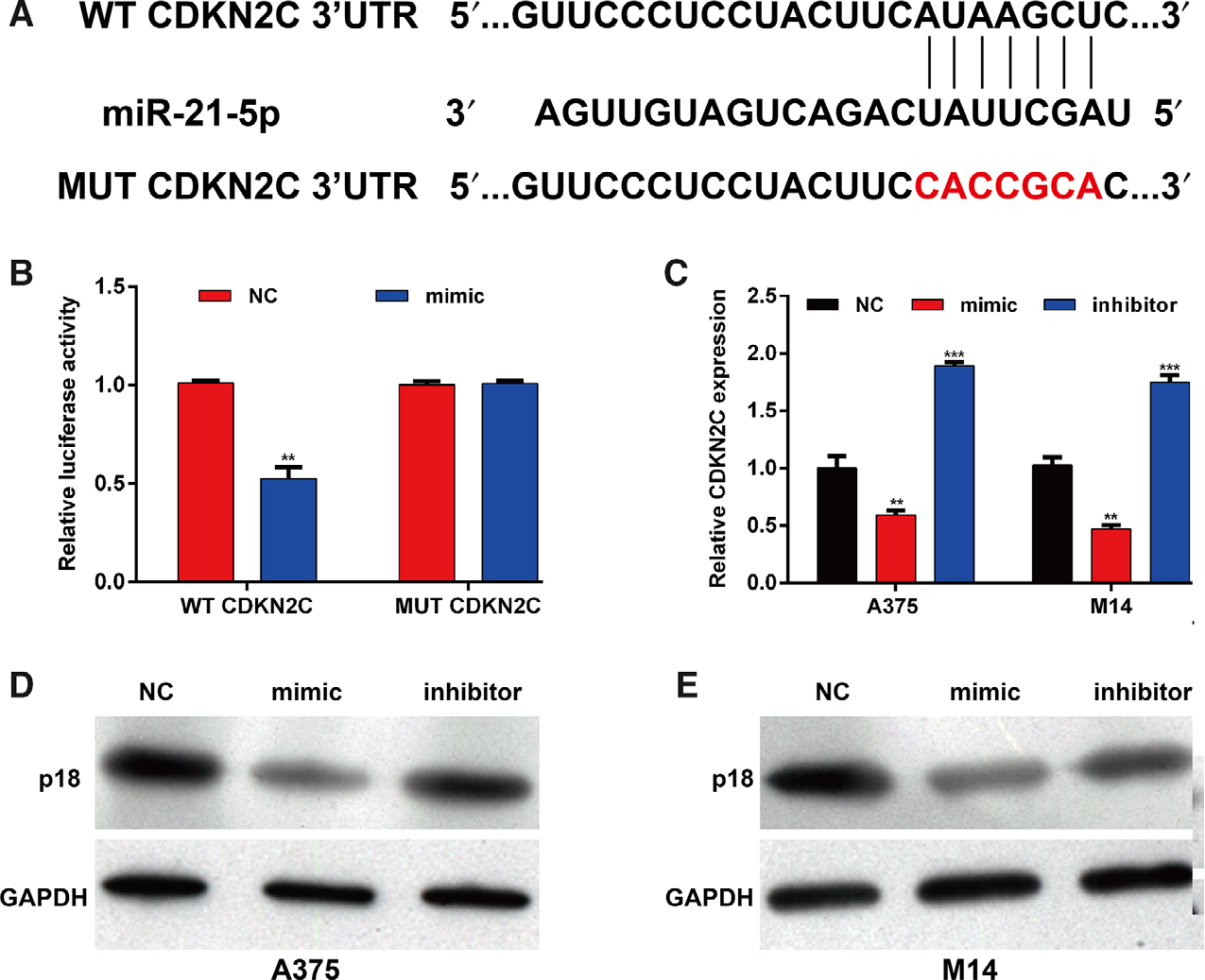
*CDKN2C* may be a direct target of miR‐21‐5p. (A) The potential binding sequences of *CDKN2C*3ʹ UTR and miR‐21‐5p based on bioinformatics analysis. The WT and mutated binding sequences are shown. (B) Relative luciferase activity in HEK293T cells transfected with reporter vector containing WT *CDKN2C* binding sequence or mutated binding sequence (MUT CDKN2C), along with miR‐21‐5p mimic and NC. Quantitative real‐time PCR analysis (C) and western blot analysis (D, E) of *CDKN2C* mRNA and protein in A375 and M14 cells transfected with miR‐21‐5p mimic, inhibitor and NC; each sample was analyzed three times. Data were expressed as mean ± SD. Differences were evaluated using one‐way ANOVA, followed by Dunnett’s test. **P < 0.01, ***P < 0.001, compared with NC.

To further investigate the molecular mechanisms of miR‐21‐5p, we predicted the potential target genes of miR‐21‐5p. Among these candidate targets, *CDKN2C* associated with cell‐cycle regulation was selected as a potential target of miR‐21‐5p. As presented in Fig. 3A, the seed sequence of miR‐21‐5p is complementary to the 3′ UTR of CDKN2C and is highly conserved. Subsequently, luciferase reporter assay was carried out to further confirm the correlation between miR‐21‐5p and *CDKN2C*. As expected, miR‐21‐5p mimic transfection significantly reduced the WT *CDKN2C* 3′ UTR luciferase activity but did not strikingly affect the MUT *CDKN2C* 3′ UTR luciferase activity (Fig. 3B). Quantitative real‐time PCR analysis suggested that the expression of *CDKN2C* mRNA was significantly suppressed by overexpression of miR‐21‐5p, whereas higher expression of CDKN2C mRNA was found after miR‐21‐5p inhibition in A375 and M14 cells (Fig. 3C). Consistently, the p18 protein expression was found to be repressed after transfection with the miR‐21‐5p mimic, but significantly elevated after transfection with the miR‐21‐5p inhibitor in A375 (Fig. 3D) and M14 cells (Fig. 3E). These data suggest that *CDKN2C* might be a direct target of miR‐21‐5p in melanoma cells.

### Overexpression of *CDKN2C* significantly attenuated the effects of miR‐21‐5p overexpression on melanoma cells

**Fig. 4:**
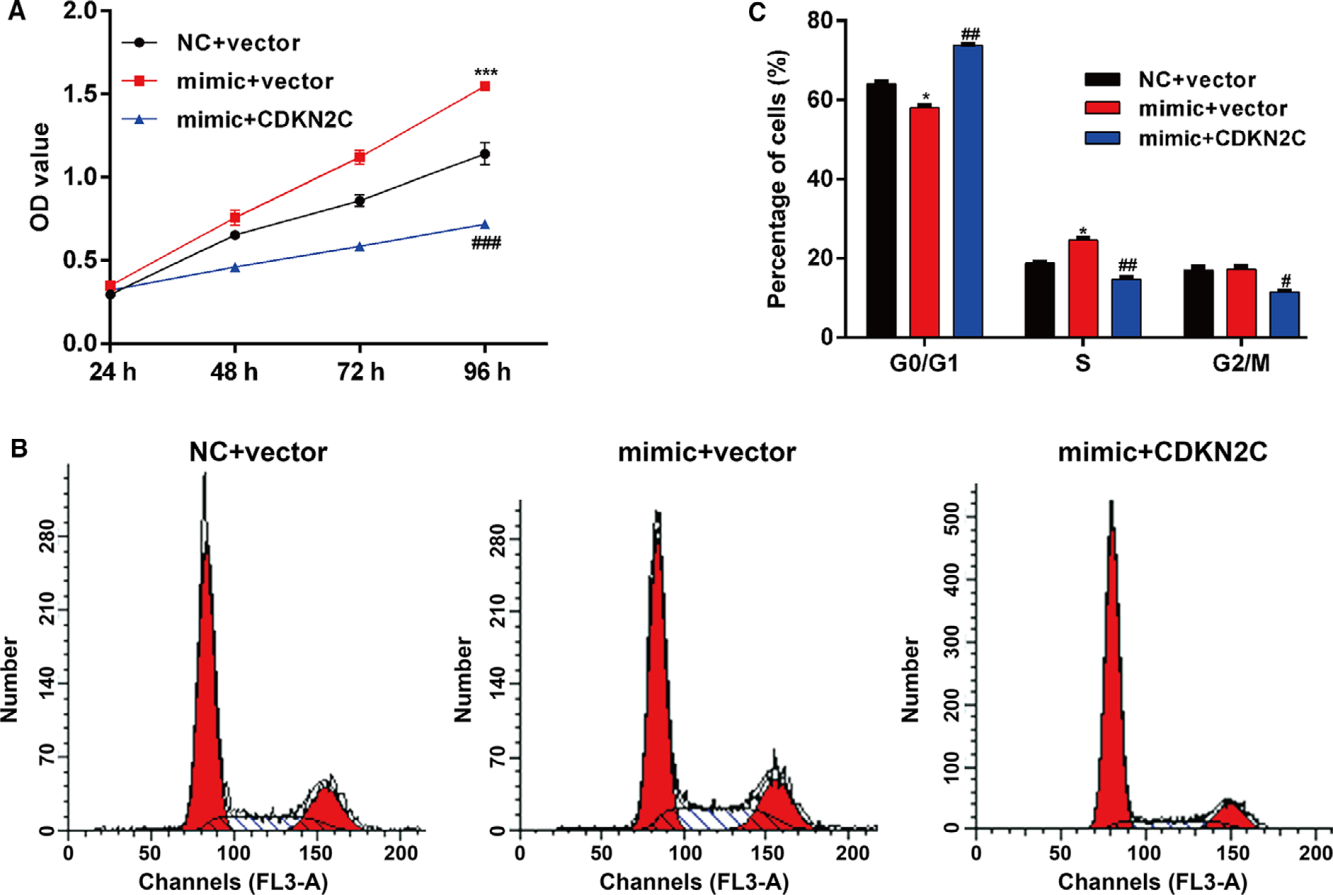
Addition of *CDKN2C* could attenuate the effects of miR‐21‐5p overexpression on melanoma cells. A375 cells were transfected with miR‐21‐5p mimic together with empty vector or pcDNA‐CDKN2C. (A) Cell proliferation was measured by the CCK‐8 assay. The CCK‐8 assay was performed every 24 h for 4 days. (B, C) Flow cytometry was performed to detect cell‐cycle distribution. Each sample was analyzed at least three times. Data were expressed as mean ± SD. Differences were evaluated using one‐way ANOVA, followed by Tukey’s test. *P < 0.05, ***P < 0.001, compared with NC + vector; ^#^ *P* < 0.05, ^##^ *P* < 0.01, ^###^ *P* < 0.001, compared with mimic + vector.

To investigate whether *CDKN2C* was a downstream functional factor involved in miR‐21‐5p regulating cell proliferation and cell‐cycle G1/S transition in melanoma, we performed rescue experiments by cotransfecting miR‐21‐5p mimic and pcDNA‐CDKN2C into A375 cells. After 48‐h transfection, we first evaluated the proliferative capacity of A375 cells using CCK‐8 assay. As a result, the enhanced cell proliferative capacity of A375 by miR‐21‐5p mimic transfection solely was remarkably attenuated by *CDKN2C* overexpression (Fig. 4A). Furthermore, the percentage of cells at G0/G1 phase was obviously decreased in A375 cells cotransfected with miR‐21‐5p mimic and empty pcDNA vector, which was significantly reversed by cotransfection with miR‐21‐5p mimic and pcDNA‐CDKN2C plasmid (Fig. 4B,C). The results imply that addition of *CDKN2C* could significantly attenuate the impact of miR‐21‐5p overexpression on cell proliferation and cell‐cycle G1/S transition.

### Knockdown of *CDKN2C* notably abolished the effects of miR‐21‐5p down‐regulation on melanoma cells

**Fig. 5:**
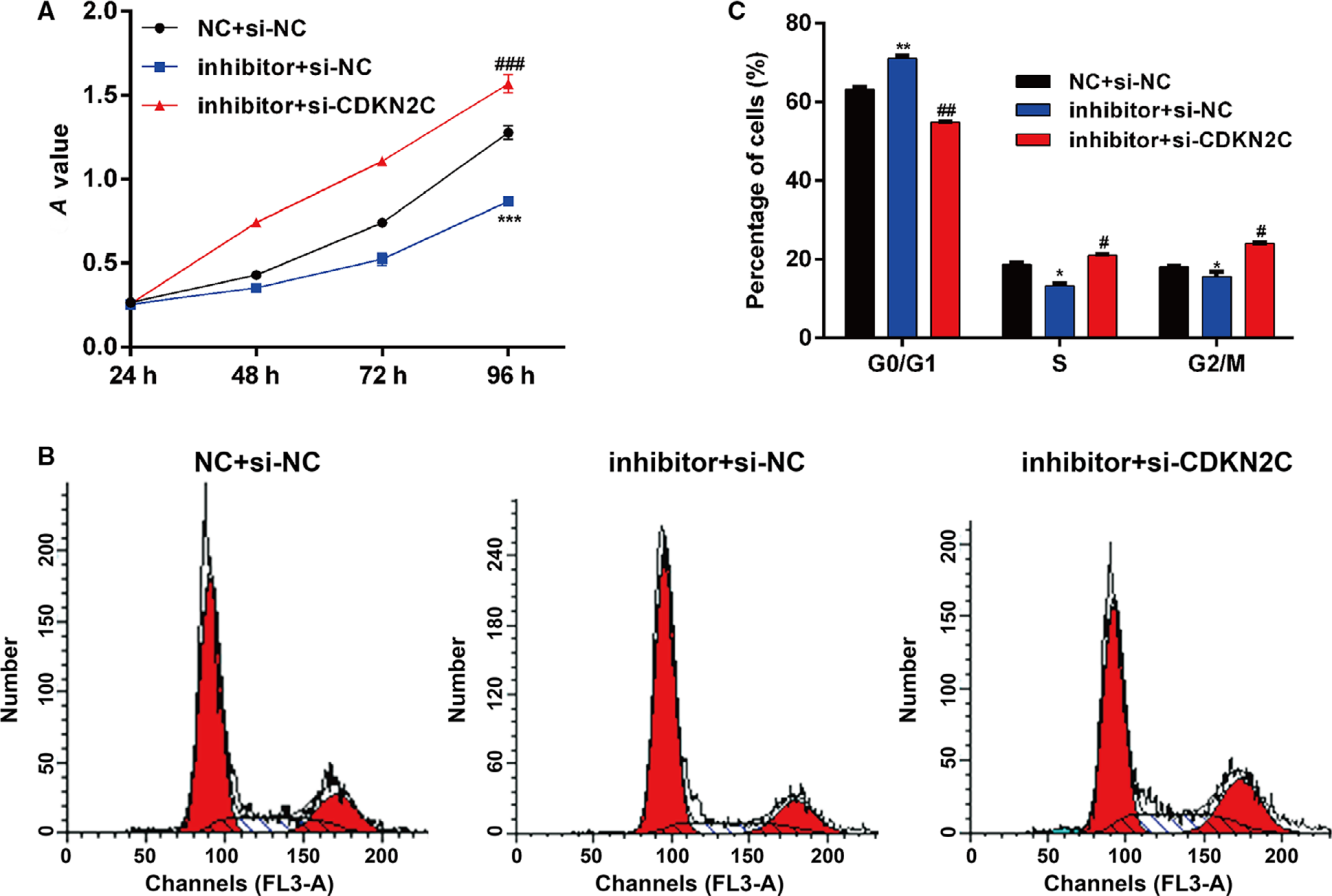
Knockdown of *CDKN2C* could abolish the effects of miR‐21‐5p down‐regulation on melanoma cells. A375 cells were transfected with miR‐21‐5p inhibitor together with si‐NC or si‐CDKN2C. (A) Cell proliferation was measured by the CCK‐8 assay. The CCK‐8 assay was performed every 24 h for 4 days. (B, C) Flow cytometry was performed to detect cell‐cycle distribution. Each sample was analyzed at least three times. Data were expressed as mean ± SD. Differences were evaluated using one‐way ANOVA, followed by Tukey’s test. *P < 0.05, **P < 0.01, ***P < 0.001, compared with NC + si‐NC; ^#^ *P* < 0.05, ^##^ *P* < 0.01, ^###^ *P* < 0.001, compared with inhibitor + si‐NC.

To further improve the earlier rescue experiments, we transfected A375 cells with miR‐21‐5p inhibitor together with si‐NC or si‐CDKN2C, followed by functional analysis. As shown in Fig. 5A, a significantly lower proliferative rate was found in A375 cells cotransfected with miR‐21‐5p inhibitor and si‐NC than that in cells cotransfected with NC and si‐NC. However, *CDKN2C* knockdown reversed the impaired cell proliferation ability induced by miR‐21‐5p down‐regulation. Similarly, cell‐cycle G0/G1 phase induced by miR‐21‐5p inhibitor transfection was abolished by *CDKN2C* knockdown in A375 cells (Fig. 5B,C). The data further demonstrated that miR‐21‐5p promoted cell proliferation and cell‐cycle G1/S transition by directly down‐regulating *CDKN2C* in melanoma cells.

## Discussion

In this study, we observed that the expression of miR‐21‐5p is up‐regulated in melanoma tissues. Consistently, increased miR‐21 expression has been observed in malignant melanocytic skin tissues compared with benign tumors [12, 13, 14]. Different from the study by Martin del Campo *et al*. [15], who showed enhancement of the invasiveness of melanoma cells by inhibition of tissue inhibitor of metalloproteinases 3, our functional experiments further indicated that miR‐21‐5p mimics significantly enhanced, whereas inhibitor suppressed, A375 and M14 cell proliferation. Similarly, miR‐21‐5p exerts an oncogene by promoting cell growth in colon cancer [10], ovarian cancer [11] and esophageal squamous cell carcinoma cells [20]. In addition, miR‐21‐5p increases the cisplatin resistance of non‐small‐cell lung cancer cells via modulating cell proliferation [21].

Related research suggests that cell proliferative capability is primarily controlled by the regulation of cell cycle to monitor DNA integrity [22]. In general, regulation of cell proliferation mainly occurs at the G0/G1 phase, during which cells integrate many signals, including growth factors and DNA damage, to determine whether cells enter into the S phase or exit to their resting stage [23, 24]. Next, we found that miR‐21‐5p knockdown significantly induced cell‐cycle G0/G1 arrest, which was significantly reversed by miR‐21‐5p overexpression. In agreement with our data, miR‐21‐5p inhibitor induced cell‐cycle G0/G1 phase arrest and inhibited the proliferation of colon adenocarcinoma cells [10]. Thus, we speculated that attenuation of cell proliferation by miR‐21‐5p knockdown might be ascribed to miR‐21‐5p inhibitor‐induced cell‐cycle G0/G1 phase arrest in melanoma cells.

Accumulating evidence has shown that miRNAs could target cyclin‐dependent kinase/cyclin complexes to affect the cell‐cycle G1/S transition in tumor cells. For instance, overexpression of miR‐95‐3p promotes cell‐cycle G1/S transition by directly targeting p21, a CDKI encoded by *CDKN2C* in hepatocellular carcinoma [25]. Ablation of endogenous miR‐25 restored expression of *CDKN1C* and significantly inhibited proliferation of glioma cells by promoting normal cell‐cycle progression [26]. Enhanced miR‐188 expression suppresses human nasopharyngeal carcinoma cell proliferation and cell‐cycle G1/S transition [27]. Here, we found that CDKN2C 3′ UTR has a binding site with miR‐21‐5p, and confirmed that *CDKN2C* was a direct target of miR‐21‐5p in melanoma cells. *CDKN2C* is an important cell‐cycle regulator highly related to the G1/S checkpoint [17]. Previous studies verified that *CDKN2C* as a CDKI could inhibit cell proliferation and cell‐cycle G1/S transition [28, 29]. In our experiment, knockdown of *CDKN2C* imitated, whereas its overexpression attenuated, the effects of miR‐21‐5p on melanoma cell growth and cell‐cycle G1/S transition. Similarly, cell‐cycle inhibitor *CDKN2C* has been reported to decrease cell proliferation in medullary thyroid carcinoma [30] and nasopharyngeal cancer [31]. Notably, *CDKN2C* plays a suppressive role in human melanoma as reported by Jalili *et al.* [32]. The facts indicated that miR‐21‐5p may promote melanoma cell proliferation and cell‐cycle progression via down‐regulating *CDKN2C*. Of course, some limitations appeared in our study, including the relevant results *in vivo*, miR‐21‐5p/CDKN2C‐mediated downstream signaling pathway and the clinical significance of miR‐21‐5p/CDKN2C in patients with melanoma, which will be further explored in the next work.

In summary, this study demonstrated that miR‐21‐5p was up‐regulated in melanoma, which accelerates the malignant progression of melanoma by promoting cell proliferation and cell‐cycle G1/S transition through targeting CDKN2C. Our preliminary results suggest that the miR‐21‐5p/CDKN2C axis might be a potential therapy in future melanoma treatment.

## Conflict of interest

The authors declare no conflict of interest.

## Author contributions

SK participated in the design of the study and provided experimental conditions. ZY and BL performed this experiment, participated in the interpretation of data and drafted the manuscript. XX analyzed the data and wrote the manuscript. All authors read and approved the final manuscript.
